# A Euploid Line of Human Embryonic Stem Cells Derived from a 43,XX,dup(9q),+12,-14,-15,-18,-21 Embryo

**DOI:** 10.1371/journal.pone.0140999

**Published:** 2015-11-05

**Authors:** Simone Aparecida Siqueira Fonseca, Roberta Montero Costas, Mariana Morato-Marques, Silvia Costa, Jose Roberto Alegretti, Carla Rosenberg, Eduardo Leme Alves da Motta, Paulo C. Serafini, Lygia V. Pereira

**Affiliations:** 1 National Laboratory of Embryonic Stem Cell (LaNCE), University of São Paulo, São Paulo, Brazil; 2 Department of Genetics and Evolutionary Biology, University of São Paulo, São Paulo, Brazil; 3 Huntington—Center of Human Reproduction, São Paulo, Brazil; Hospital Authority, CHINA

## Abstract

Aneuploid embryos diagnosed by FISH-based preimplantation genetic screening (PGS) have been shown to yield euploid lines of human embryonic stem cells (hESCs) with a relatively high frequency. Given that the diagnostic procedure is usually based on the analysis of 1–2 blastomeres of 5 to 10-cell cleavage-stage embryos, mosaicism has been a likely explanation for the phenomena. However, FISH-based PGS can have a significant rate of misdiagnosis, and therefore some of those lines may have been derived from euploid embryos misdiagnosed as aneuploid. More recently, coupling of trophectoderm (TE) biopsy at the blastocyst stage and array-CGH lead to a more informative form of PGS. Here we describe the establishment of a new line of hESCs from an embryo with a 43,XX,dup(9q),+12,-14,-15,-18,-21 chromosomal content based on array-CGH of TE biopsy. We show that, despite the complex chromosomal abnormality, the corresponding hESC line BR-6 is euploid (46,XX). Single nucleotide polymorphism analysis showed that the embryo´s missing chromosomes were not duplicated in BR-6, suggesting the existence of extensive mosaicism in the TE lineage.

## Introduction

Human embryonic stem cells (hESCs) are pluripotent cells derived from the inner cell mass of blastocysts, and a potential source of tissue for cell therapy as well as for basic research on different aspects of human development [1,2]. Usually, lines of hESCs are derived from surplus embryos produced for reproductive reasons. Although most lines have been established from normal/good quality embryos, hESC derivation has been achieved also from morphologically abnormal embryos [3–5], and from embryos scored as aneuploid by FISH analysis of cleavage-stage blastomeres [6–8]. Surprisingly, up to two thirds of the lines established from the latter turned out to be euploid [9]. Genetic analysis of the corresponding cell lines show that instead of extrusion or duplication of aneuploid chromosomes during cell line establishment, leading to embryo-self correction, mosaicism in the original embryo is the most likely explanation for this phenomenon in most instances (reviewed by [10]).

However, a series of studies have questioned the value of FISH-based PGS based on findings of euploidy in blastocysts diagnosed as aneuploid by FISH at cleavage-stage [11–14]; and on lack of clinical benefit of the procedure in randomized trials (reviewed by [15]). Therefore, there is a possibility that misdiagnosis of aneuploidy in the original embryo explains at least some of the resulting euploid lines of hESCs.

More recently, SNP-based array CGH has been introduced in PGS as an alternative to FISH analysis. Coupled with trophectoderm biopsy at the blastocyst stage, the technique was shown to be a more robust method of genetic screen in the human preimplantation embryo [16,17]. Here we describe the establishment of lines of hESCs from embryos diagnosed as aneuploid by array-CGH of TE, and report the derivation of a hESC line from a blastocyst with a complex chromosomal content, 43,XX,dup(9q),+12,-14,-15,-18,-21. SNP analysis indicates that the euploid cells were already present in the original embryo.

## Materials and Methods

### Embryo biopsy and array-CGH

Embryos were cultured in Global Medium (Life Global, Guilford, CT, USA) until day-5 or 6 (D5 or D6) after *in vitro* fertilization by intracytoplasmic sperm injection (ICSI), in a humidified atmosphere of 5% CO_2_, 5% O_2_ and 90% N_2_ at 37°C. Embryos that reached the blastocyst-stage were biopsied. Drilling in zona pellucida was made by laser at day-3. Four to eight trophoblast cells were biopsied by aspiration on day 5/6. The cells were washed in PBS, collected into sterile PCR tubes. DNA extraction and amplification were performed by SurePlex kit, according to the manufacturer’s instructions (Illumina, San Diego, CA, USA).

The final wash medium was used as negative control and normal male genomic DNA as positive control. After the preamplification assay, samples were labeled using Fluorescent Labeling System (dCTP, Cy3 –DNA sample, and Cy5—DNAref control, Illumina). The array-CGH was performed with 24sure (v3.0) slides (Illumina) according to manufacturer’s instructions.

Embryos were cryopreserved by vitrification as described [18]. Embryos diagnosed as aneuploid were donated for research according to the Brazil´s Bio-safety Law 11.105—March 25, 2005, with written informed consents signed by biological parents, and the approval of the Ethics Committee of the Bioscience Institute of the University of São Paulo (http://www.ib.usp.br/formularios.php?menu=1—protocol number 044/2006).

### Derivation and culture and of hESCs

Embryos were thawed and cultured overnight in Global Medium (Life Global) supplemented with 10% of serum substitute supplement (Ingamed, Maringa, PR, Brazil) covered by oil for Embryo Culture (Irvine Scientific, CA, USA) at 37°C in a humid and atmospheric control incubator (5% CO2 and 5% O2).

To establish the hESC lines, embryos were washed quickly in Tyrode's Solution—Acidified (Irvine Scientific, CA, USA) to remove the zona pellucida (ZP) and were immediately washed in medium with Hepes (GV Hepes, Ingamed), under stereomicroscope (Nikon). To derive new hESC lines under defined xeno-free culture condition, we used the CloneStem kit (Biolamina, Sweeden) that contain recombinant 521 laminin and E-cadherin as matrix, and E8 medium (Invitrogen, Grand Island, NY, USA) as described [19]. Embryos freed of ZP were plated individually in plates pretreated with Laminin 521 and E-cacherin (Biolamina, Sweden) according to the manufacturer’s instructions, and were cultured in E8 medium (Invitrogen) supplemented with 10% human albumin serum (Biolamina) at 37°C in controlled atmosphere (5% CO2 and 5% O2) for 48h. After this period, the medium was changed daily.

The first passage was made mechanically after 15 days and the fragments were transferred to a new similar area, pretreated with laminin 521 and e-cadherin, in E8 medium supplemented with 5 uM of Rock Inhibitor (Y27632, StemGent,Cambridge, MA, USA). The medium was changed every day. The next three passages were made mechanically and the fragments were transferred to plates pretreated only with Laminin 521 (Biolamina) in E8 medium, in a split ratio of 1:2. After passage five, the hESCs were maintained in Geltrex and E8 medium (LifeTechnologies, CA, USA), and passaged with Gentle Cell Dissociation (Stem Cell technologies, Vancouver, Canada).

### Immunocytochemistry

Cells at passage 10 were cultivated in slide chambers (Labtech, NUNC, Roskilde, Denmark) and were fixed and immunostained according standard protocols [20]. The cells were incubated overnight at 4°C with primary antibodies at the dilutions 1:100 (OCT3/4 (Santa Cruz Biotechnology, Dallas, TX, USA)), 1:100 (SSEA4 (StemGent)) and 1:25 (Nanog (R&DSystems, Minneapolis, MN, USA)). After wash, a new incubation with secondary antibodies conjugated with Alexa-Fluor 488 or 594 (Molecular Probes, Life Technologies) at 1:1000 dilutions was performed. Slides were mounted in VectaShield mounting medium containing DAPI (Vector laboratories, Burlingame, CA, USA). The analysis was done using an Axiophot 2 (Carl Zeiss, Germany) epifluorescent microscopy and images were captured by CCD camera using the ISIS software (MetaSystem, Altlusshein, Germany).

### Flow cytometry

Cells were fixed and labeled using *Human Pluripotent Stem Cell Transcription Factor Analysis Kit* (BD, San Diego, CA, USA) according to the manufacturer’s instructions. Cells were incubated with human monoclonal antibodies specific to Nanog-PE, Oct3/4-PerCP-Cy5.5 and Alexa 647-Sox2, and corresponding isotype. Fifty thousand events were acquired in *BD Accuri C6 flow cytometer* using the kit template. Cells were gated on light scatter properties and analyzed for expression of key pluripotency transcription factors using *BD Accuri C6 software*.

### Cytogenetic analysis

Cells at passage 10 were subjected to standard G-banding karyotype analysis as described [21]. At least 50 metaphases were analyzed in order to detect the presence of mosaicism.

DNA from undifferentiated cells at passage 4 and 20 was used to perform array-CGH using the whole genome Cytosure^™^, ISCA V2 array 4X180K (Oxforg Gene Technology, OGT, UK) containing ~180.000 oligonucleotides. Briefly, samples were labeled with Cy3- and Cy5-dCTPs by Cytosure Genomic DNA Labelling *kit* (OGT, Oxford, UK) and hybridization by *Agilent Oligo a-CGH Hybridization kit* (*Agilent Technologies*, Califórnia, USA). Scanned images of the arrays were processed and analyzed using Feature Extraction and Genomic Workbench softwares (both from Agilent Technologies), using the statistical algorithm ADM-2 and a sensitivity threshold of 6.0. We used two reversed labeled hybridizations for each sample. Gains or losses in copy number were accepted when the log2 ratio of the Cy3/Cy5 intensities of a given genomic segment was > 0.6 or < -0.8, respectively, and where putative alterations encompassed at least three consecutive probes; any alterations not detected in both dye-swap experiments were disregarded. All detected imbalances were compared to CNVs reported in the Database of Genomic Variants (DGV; http://projects.tcag.ca/variation/—freeze of March, 2011), and to data from our own group.

### SNP genotyping

SNP genotyping was performed using the CytoSNP 850K BeadChip (Illumina) which contains 850,000 SNP probes covering the whole-genome, with enriched coverage for 3,262 well-known genes of constitutional and cancer diseases (The International Collaboration for Clinical Genomics; http://www.iccg.org/). Labeling, hybridization, and washing procedures followed the manufacturer’s instructions. Microarray slides were scanned using the iScan System (Illumina), and the gtc files were loaded on the BlueFuse Multi Software v3.2 (BlueGnome) to evaluate experimental quality and to SNP calling.

The SNP and copy number data from BR6_P21 (file name 9993049081_R07C01.gtc) and BR6_P4 (file name 9993049081_R08C01.gtc) can be accessed at http://dx.doi.org/10.7910/DVN/DYGXPG. Files can be read in the Bluefuse program, freely available from Illumina.

### In vitro differentiation

Spontaneous differentiation of the BR-6 hESCs was performed as described [22]. Briefly, cell aggregates were grown in suspension in non-adherent plates covered with 1% agarose (Invitrogen, Lifetechnologies) in differentiation medium containing DMEM high glucose (Invitrogen), 20% FBS ES qualified (Invitrogen), 1% penicillin-streptomicin (Invitrogen), 1X Glutamax (Invitrogen). After one week in suspension, embryoid bodies (EBs) were transferred to adherent plates precoated with 0.1% gelatin (Stem cell Technologies), and grown for additional 8–10 days.

Differentiated cells were harvested with Trizol (Life Technologies) and RNA extraction was performed using RNeasy kit (Qiagen, Hilden, Germany). One microgram of RNA was used for subsequent reverse transcriptase reactions using *High Capacity cDNA* kit (Life Technologies). Pluripotency and trilineage differentiation potential was assessed using the TaqMan^®^ hPSC Scorecard^™^ kit 96w following manufacturer’s instructions and run on a *StepOne Plus System* (Applied Biosystems, Life Technologies) Data analysis was performed using the cloud based TaqMan^®^ hPSC Scorecard^™^ analysis software (Applied Biosystems, Life Technologies

## Results

### Array-CGH and hESC line derivation

**Fig 1:**
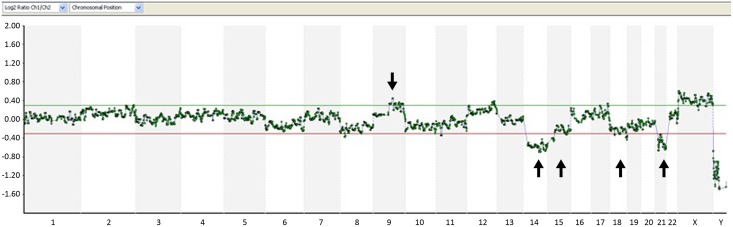
Array-CGH analysis of TE biopsy from embryo 1. Arrows point to the regions of aneuploidy. Increased X and decreased Y signals are due to the use of 46,XY control DNA.

Array-CGH analysis identified 20 aneuploid embryos that were subsequently donated for hESC line derivation. Following thawing, all embryos were cultured overnight. Ten embryos degenerated, and the remaining 10 were used for hESC derivation under defined xeno-free culture condition (Table 1, Fig 1). Two embryos (embryos 1 and 10) attached to the culture plate and presented cell growth. From these, despite its complex aneuploidy, only embryo 1 gave rise to a new line of hESC, named BR-6 (Fig 1). This rate of derivation, although relatively low, was similar to those obtained by our group with euploid embryos [24, 25].

**Table 1:** Embryos plated for hESC derivation.

Id	CGH-array	Morphology* (after thawing)	Morphology* (overnight culture)	hESC
**1**	43, XX, +9q,+12,-14,-15,-18,-21	4CC	4BC	BR6
**2**	45, XX, -6	3CC	3CC	-
**3**	46, XX,-9,-10,+13,+17	4CC	4 (collapsed)	-
**4**	47, XX, -7, +11,+21	4CC	5CC	-
**5**	47, XX, +18	4CC	4CC	-
**6**	46, XY, -1p	3CC	5BB	-
**7**	46, XY, +8,-21	3CC	4CC	-
**8**	47, XY, +1p,+22	4CC	5CB	-
**9**	45, XY, -15	4CC	5CC	-
**10**	45, XY, -21	3CC	5BB	-

*According to [23]

**Fig 2:**
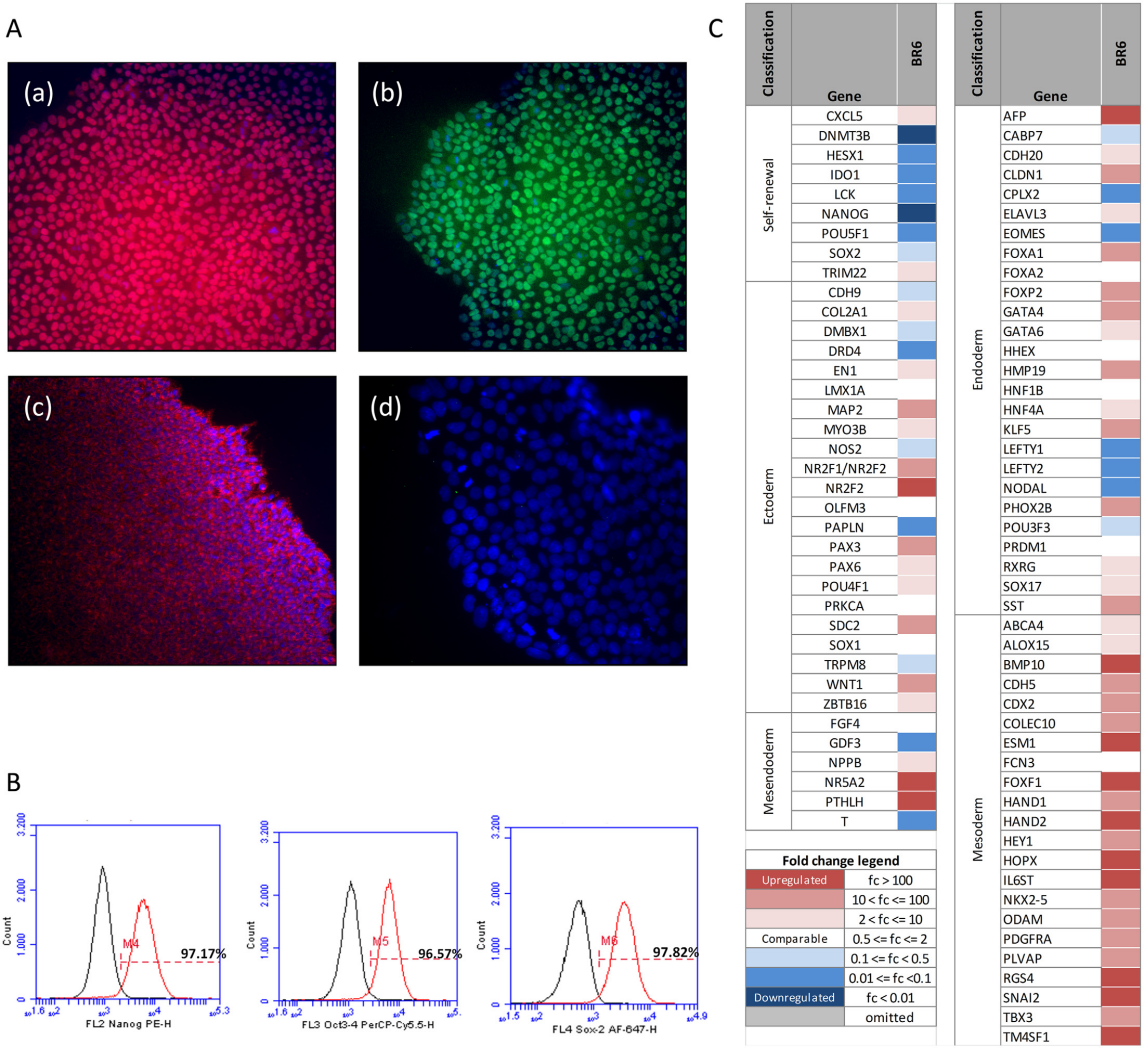
Characterization of pluripotency of BR-6. (A) Immunofluorescence using antibodies against (a) OCT4 in red, (b) NANOG in green and (c) SSEA-4 in red. Nuclei were counterstained with (d) DAPI.; (B) Flow cytometry showing cells positive for NANOG, OCT3/4 and SOX2 (red curves). Black curves correspond to cells with control isotypes. Percentage of positive cells are indicated; (C) ScoreCard results: change in expression levels of genes characteristic of self-renewal and of each germ layer in embryoid bodies in relation to hESCs are indicated by color code (from red–upregulation, to blue–downregulation. See “Fold change legend”).

Immunofluorescence of undifferentiated BR-6 cells showed that they are positive for the pluripotency markers OCT4, NANOG and SSEA4 (Fig 2A). Analysis by flow cytometry confirmed the findings for OCT4 and NANOG, and showed up to 97.8% of SOX2 positive cells (Fig 2B).

Finally, Scorecard analysis of the differentiation of day 15 BR-6 embryoid bodies showed that, although able to express markers from the three embryonic germ layers, the cell line has a propensity for differentiation into mesoderm (Fig 2C).

### Karyotype and array-CGH analysis of BR-6

**Fig 3:**
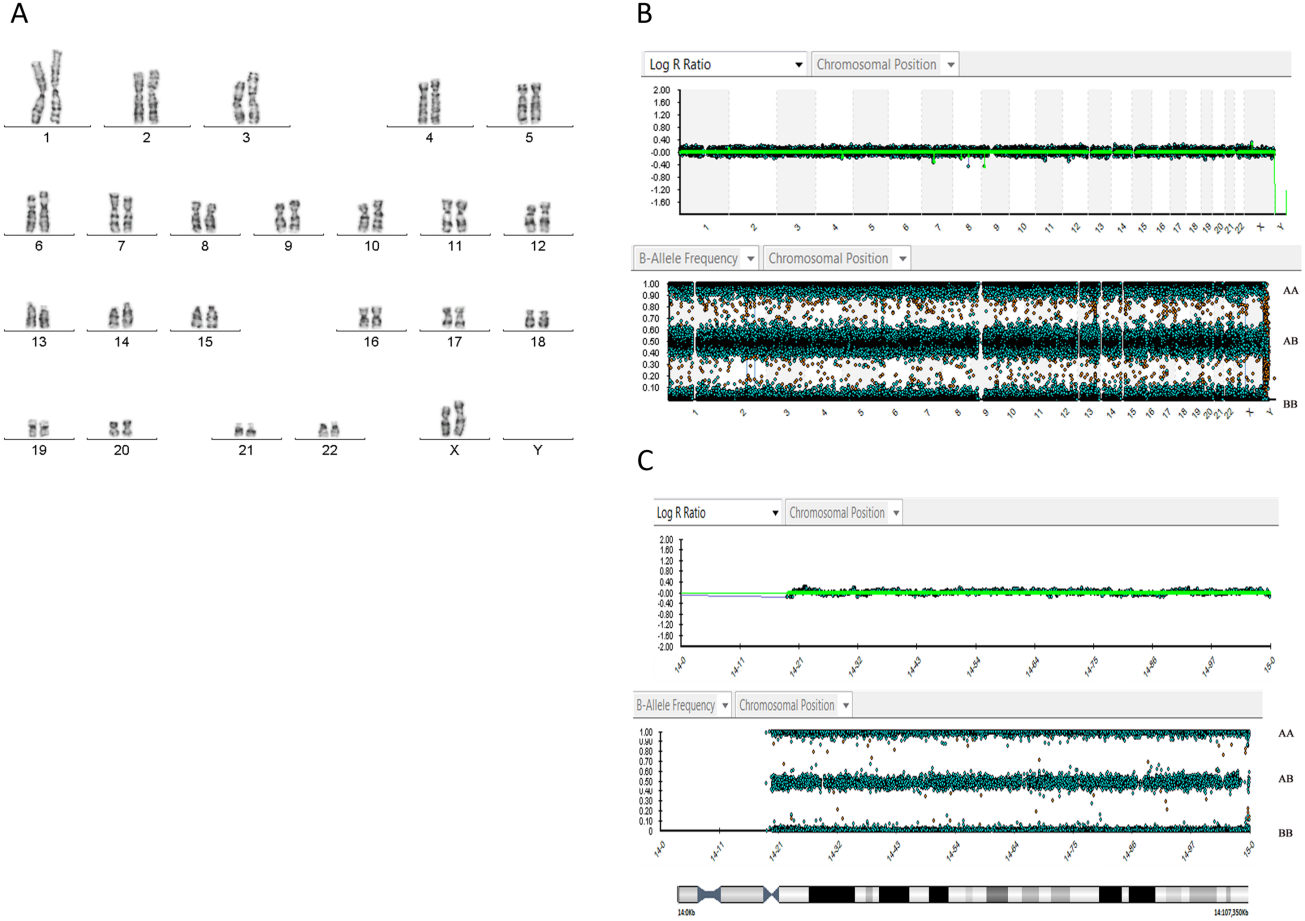
Genomic analysis of BR-6. (A) G-banding karyotype; (B) Copy number (above) and SNP (below) array profiles of all chromosomes from BR6, showing that neither aneuploidies nor large homozigous segments are present; (C) Detailed data of copy number (above) and SNP (below) array profiles of chromosome 14, showing no copy number alterations and heterozigosity of several SNPs throughout the chromosome, ruling out duplication of that chromosome.

Standard G-banding karyotype of 50 metaphases of BR-6 revealed a normal 46,XX chromosomal constitution, without any indication of gain or loss of chromosome segments (Fig 3A). More detailed analysis of the chromosomal content of BR-6 was performed by array-CGH analysis, which confirmed a normal chromosome balance in this line of hESC (Fig 3B).

In order to determine whether the normal karyotype of BR-6 was a result of self-correction, we performed SNP-array analysis (Fig 3C)–self correction would lead to complete homozygocity of the originally aneuploid chromosomes. Genotyping results showed extensive heterozygocity along the whole genome. In particular, there was no indication of duplication of chromosomes 14, 15,18 or 21, which would be required for the correction of the monosomies present in embryo 1. These results indicate that despite the complex aneuploidy detected in the TE, embryo 1 had enough euploid cells to give rise to a normal diploid line of hESCs.

## Discussion

Euploid lines of hESCs have been established from embryos scored as aneuploid by FISH-PGS at a significantly high rate (reviewed by [10]). However, the effectiveness of the genetic screening technique has been challenged, which may suggest that a fraction of those embryos were originally euploid. More recently, the combination of improved *in vitro* conditions for the culture of human embryos to the blastocyst stage, biopsy of 6–10 cells from the TE, and array-CGH analysis has lead to a more robust strategy for PGS [26]. Indeed, one study found that 58% of blastocysts scored at cleavage-stage as aneuploid by FISH analysis were completely euploid by subsequent SNP-array CGH analysis [17]. Of these, none presented uniparental disomy of the originaly aneuployd chromosome, arguing against monosomy and trisomy rescue during development from cleavage to blastocyst stage, thus supporting the idea of mosaicism in the cleavage-stage embryo.

In addition, Treff et al. [16] compared FISH and array-CGH results of chromosomal content of blastomeres from the same embryos at the same developmental stage. Their data showed that, while FISH detected aneuploidies to be present on 100% of the embryos, array-based analysis of cells of the same embryos found aneuploidies in only 39% and uniform diploidy in 61% of embryos, suggesting that FISH-PGS overestimates aneuploidies at the cleavage-stage.

Thus, it is reasonable to propose that a significant portion of the aneuploid embryos reported to yield euploid lines of hESCs were misdiagnosed and originally diploid rather than mosaics. In the present study, we worked with human blastocysts vitrified after TE biopsy, and scored as aneuploid by array-CGH. Therefore, the emergence of a diploid hESC line is more likely to be due to mosaicism of the blastocyst or self-correction than to misdiagnosis. However, self-correction of the complex aneuploidy is very unlikely, and was actually discarded by SNPs analysis of BR-6. Therefore, preferential segregation of the aneuploidy to the TE lineage, as proposed by others (reviewed in [27]), or growth/viability advantage of euploid cells from the inner cell mass could have led to the emergence of a diploid cell line.

To our knowledge, this is the first euploid hESC line derived from an embryo diagnosed at the blastocyst stage by array-CGH with a complex aneuplody, including 4 monosomies, reinforcing the value of unviable human embryos for the field of cell therapy.
